# Development and validation of an algorithm to assess risk of first-time falling among home care clients

**DOI:** 10.1186/s12877-019-1300-2

**Published:** 2019-10-14

**Authors:** Ayse Kuspinar, John P. Hirdes, Katherine Berg, Caitlin McArthur, John N. Morris

**Affiliations:** 1 School of Rehabilitation Science, McMaster University, 1400 Main St. W. Room 435, IAHS, Hamilton, ON L8S 1C7 Canada; 2 School of Public Health and Health Systems, University of Waterloo, Waterloo, ON Canada; 3 Department of Physical Therapy and Rehabilitation Sciences Institute, University of Toronto, Toronto, ON Canada; 4 GERAS Centre for Aging Research, McMaster University, Hamilton, ON Canada; 5 Hebrew Senior Life, Institute for Aging Research, Boston, MA USA

**Keywords:** Falls, Home care, Older adults, Machine learning, interRAI

## Abstract

**Background:**

The falls literature focuses on individuals with previous falls, so little is known about individuals who have not experienced a fall in the past. Predicting falls in those without a prior event is critical for primary prevention of injuries. Identifying and intervening before the first fall may be an effective strategy for reducing the high personal and economic costs of falls among older adults. The purpose of this study was to derive and validate a prediction algorithm for first-time falls (1^st^Fall) among home care clients who had not fallen in the past 90 days.

**Methods:**

Decision tree analysis was used to develop a prediction algorithm for the occurrence of a first fall from a cohort of home care clients who had not fallen in the last 90 days, and who were prospectively followed over 6 months. Ontario home care clients who were assessed with the Resident Assessment Instrument-Home Care (RAI-HC) between 2002 and 2014 (*n* = 88,690) were included in the analysis. The dependent variable was falls in the past 90 days in follow-up assessments. The independent variables were taken from the RAI-HC. The validity of the 1^st^Fall algorithm was tested among home care clients in 4 Canadian provinces: Ontario (*n* = 38,013), Manitoba (*n* = 2738), Alberta (*n* = 1226) and British Columbia (*n* = 9566).

**Results:**

The 1^st^Fall algorithm includes the utilization of assistive devices, unsteady gait, age, cognition, pain and incontinence to identify 6 categories from low to high risk. In the validation samples, fall rates and odds ratios increased with risk levels in the algorithm in all provinces examined.

**Conclusions:**

The 1^st^Fall algorithm predicts future falls in persons who had not fallen in the past 90 days. Six distinct risk categories demonstrated predictive validity in 4 independent samples.

## Introduction

Falls are a major public health concern because 28 to 35% of older adults over the age of 65 fall each year [1]. This problem is not new, but it remains one of the most complicated, high-risk and high-cost unsolved issues that the healthcare system faces. Falls are the principal cause of injury-related hospitalizations among older adults and are linked with the longest length of stay compared to all other causes [2]. In addition to injury, hospitalization and disability, falls can have significant negative consequences on the mental well-being of older adults [3]. Considering the substantial personal and economic impact of falls [1] and the rapidly increasing number of older adults in most nations, [4] fall risk assessment and prevention is a global healthcare priority.

Regardless of the tool used for identifying fallers, the strongest single indicator and the most frequently used factor for fall prediction is history of falling [5, 6]. The risk for falling is three times higher for persons who have fallen before compared to those who have not [6, 7]. Although fall history is a major predictor of future falls, it only provides information about persons who are repeat fallers and has no preventive value for persons with an impending first-time fall event.

Several studies developed models to predict the risk of falling in older adults, but most were based on heterogeneous samples that included individuals with a fall prior to study enrolment [8–11]. Further, several risk factors have been identified to predict falls, but few studies examined whether the factors interact with one another to produce hierarchical patterns of risk. To address this gap, machine learning tools can provide a simple visual representation of complex non-linear associations to identify at-risk sub-groups. They optimize prediction of a target variable (i.e. falls) by recursively dividing subjects into different subgroups so that members within each subgroup are as homogeneous as possible yet distinct from members in other groups [12].

This study involved the derivation and validation of a prediction algorithm for first-time falls (1^st^Fall) among home care clients in Canada. We developed a predictive model for the occurrence of a first fall from a cohort of more than 80,000 adults who had not fallen in the last 90 days, and who were prospectively followed over 6 months. We then assessed the validity of the prediction algorithm in four different provincial samples.

## Methods

### Sample

The algorithm was developed based on data from Ontario home care clients assessed between 2002 and 2014 (*n* = 126,703). Clients with no history of falls within the past 90 days of admission (baseline), were followed prospectively until the next point of assessment (about 6 months later). The baseline sample (*n* = 126,703) was randomly split into 70% algorithm derivation (*n* = 88,690) and 30% validation (*n* = 38,013) sets.

Validation was done with Canadian home care data from Ontario (*n* = 38,013), Manitoba (*n* = 2738), Alberta (*n* = 1226), and British Columbia (*n* = 9566). Clients with no history of falls within the past 90 days of admission were followed for approximately 6 months. Ethics approval was received from the University of Waterloo’s Office of Research Ethics.

### Variables

The independent variables were taken from the Resident Assessment Instrument-Home Care (RAI-HC) [13]. The RAI-HC is a patient centered assessment system that includes items on symptoms, function and quality of life. It is administered by trained health professionals on admission to home care and at about every 6 months thereafter. The reliability and validity of the RAI-HC instrument has been established [14–16]. For example, in a large multi-national study, [15] the RAI-HC items met or surpassed standard cut-offs values for acceptable reliability and a significant percentage demonstrated excellent reliability. A number of studies have also demonstrated construct validity of the items and the embedded scales [16, 17]. All items and summary scales included in the RAI-HC were considered for algorithm derivation, including, for example, the Cognitive Performance Scale, [18] Pain Scale [19] and Activities of Daily Living Hierarchy Scale [20].

The dependent variable was falls in the past 90 days at follow-up dichotomized as 0 falls and 1 or more falls.

### Algorithm derivation

Decision tree models use decision rules to form a sequence of partitions and progressively divides the target value (i.e., fall status) into smaller and smaller homogeneous groups based on the input value [21]. They are particularly effective in complex data sets where the end result is an outcome of many interacting factors. Therefore, decision tree analysis was chosen to be the most suitable tool to carry out the predictive modeling in this paper. SAS Enterprise Miner 13.1 [22] was used to conduct the decision tree analysis.

The starting point of the decision tree is referred to as the root and the partitioning at the end are called the terminal nodes [23]. The iterative partitioning generates terminal nodes that contain the final estimated probabilities or target proportions. The logworth statistic, which is the negative log of the *p*-value for the Chi-Square test, was used for splitting branches and growing the tree. Good predictors have higher logworth values. While constructing the decision tree, each node was individually assessed for different independent variables and cut-off values in order to produce well differentiated paths with proportional sample sizes. Several different variations of the decision tree were developed interactively with selection of the final tree guided by clinical judgement and the statistical ranking of variables by Enterprise Miner. The last stage of the analysis involved combining terminal nodes with similar levels of risk to form higher-level groups.

### Algorithm validation

The algorithm was applied in four Canadian provinces that use the RAI-HC. We hypothesized that the percentage of falls would increase as the risk category increased. Logistic regression analysis was utilized to derive odds ratios to further assess the validity of the algorithm in different samples. Fall status was the dependent variable and the decision tree levels were the independent variables.

## Results

### Sample characteristics

Table 1 presents the clinical characteristics of the derivation and validation samples (four provinces). The derivation sample was predominantly female and over the age of 65 (mean age 77 ± 14 standard deviations). Approximately 34% of the sample reported living alone, 24% had bladder incontinence, and about half (53%) had unsteady gait. Almost 50% of the sample had no cognitive impairment, and 19% reported poor self-rated health. Similar rates were observed in the four validation samples.

**Table 1:** Baseline demographic and clinical characteristics of the derivation and validation samples

	Derivation Sample	Validation Samples			
	Ontario (*n* = 88,690)	Ontario (*n* = 38,013)	Manitoba (*n* = 2739)	Alberta (*n* = 1226)	British Columbia (*n* = 9568)
Characteristic	%	%	%	%	%
Age (years)					
Less than 65	17	17	13	9	7
65–79	33	32	29	29	27
80 or older	51	51	58	62	66
Female	65	65	64	62	62
Living alone	34	34	46	29	42
Instrumental Activities of Daily Living Difficulty Scale (out of 6)					
Independent, Supervision or Limited Assistance (0 to 3)	29	29	28	33	26
Extensive or total assistance (4 to 6)	71	71	72	67	74
Activities of Daily Living Hierarchy (total 6)					
Independent, Supervision or Assistance (0 to 3)	94	95	97	97	96
Extensive or total assistance in eating or locomotion (4 to 6)	6	5	3	3	4
Cognitive Performance Scale (out of 6)					
Intact (0)	47	47	31	25	18
Borderline intact to moderate impairment (1 to 3)	49	48	66	70	76
Moderately severe to very severe (4 to 6)	5	5	3	5	6
Bladder Incontinence (Incontinent episodes ≥2 per week)	24	24	20	19	24
Unsteady gait	53	53	42	42	51
Conditions or diseases that make cognition, ADL, mood, or behaviour patterns unstable	36	36	42	39	52
Parkinsonism	3	3	4	3	4
Poor self-rated health	19	19	16	14	16
Alzheimer’s or Dementia	21	21	32	46	46

*Percentages are rounded to the nearest integer*

### Fall-risk assessment algorithm derivation (1^st^Fall)

**Fig. 1:**
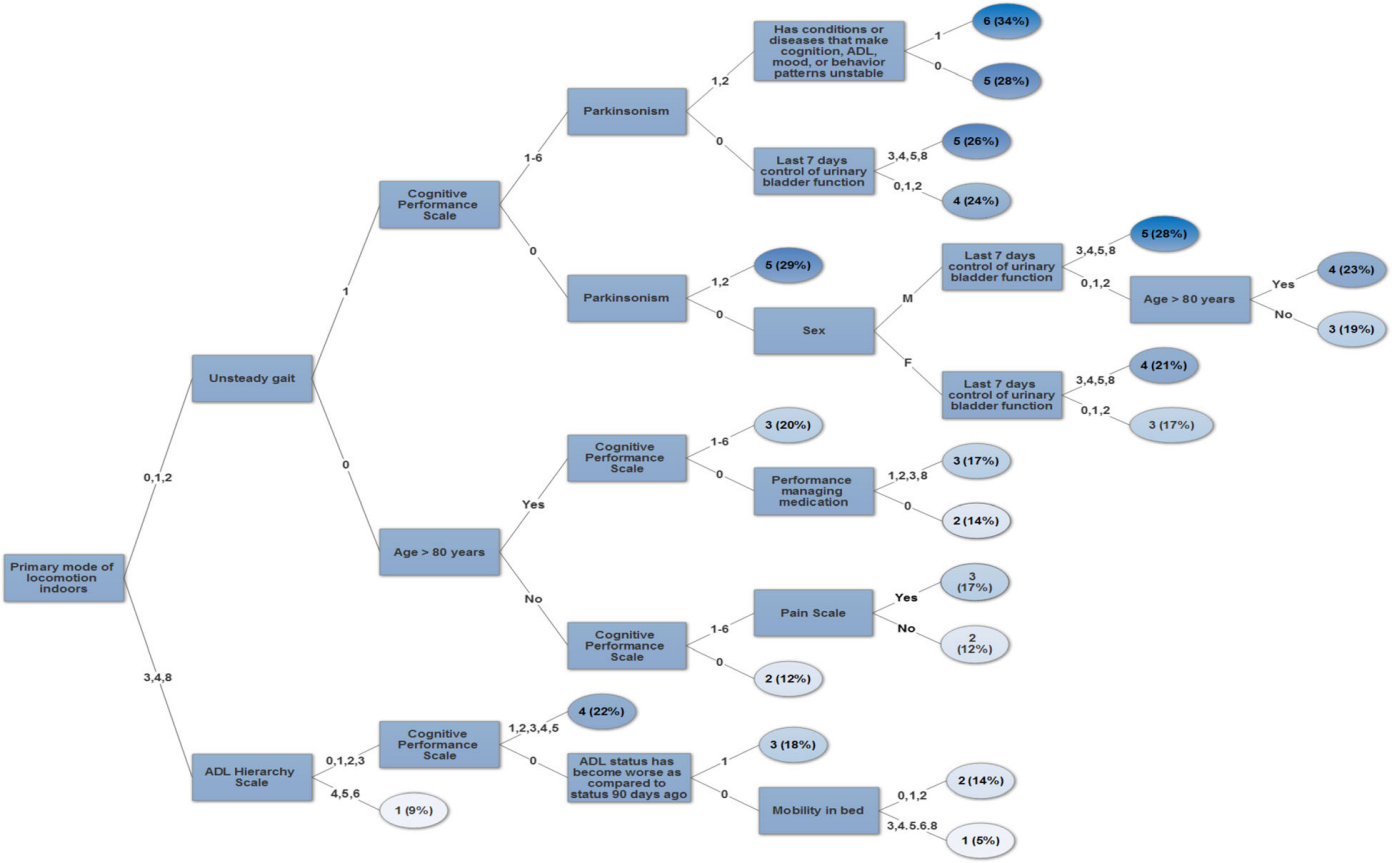
Decision tree algorithm 1^st^Fall

Figure 1 shows the logic of the decision tree algorithm (1^st^Fall), which had 21 terminal nodes with fall rates from 5 to 34%. We then identified clusters of similar risk rates and grouped them into 6 categories with rates of 5–10%, 11–15%, 16–20%, 21–25%, 26–30%, and 31–35%. About one-third of home care clients without prior falls were in the top three levels of the 1^st^Fall algorithm (Table 2). The variables used to discriminate between fall risk levels were: primary mode of locomotion indoors, unsteady gait, age, sex, Cognitive Performance Scale, Activities of Daily Living Hierarchy, Pain Scale, Parkinsonism, managing medication, bladder incontinence, worsening of activities of daily living status, unstable health patterns and mobility in bed. The root node was divided based on whether individuals were ambulatory (with or without a walking aid) or non-ambulatory indoors (use of a wheelchair or scooter). Those that were ambulatory were split further based on if they presented with unsteady gait or not. Individuals who had unsteady gait, cognitive impairment, Parkinsonism and unstable health patterns were at highest risk of having a fall. Individuals who did not present with unsteady gait but had cognitive impairment, pain and reduced performance with managing medication were at moderate risk of falling. Conversely, individuals without cognitive impairment, pain or problems with managing medication were at lower risk of falling. Fall rates were 5 and 9% for individuals in group 1 (low risk) and 34% for individuals in group 6 (high risk). The two highest risk brackets made up 11.4% of the total sample, which translates into 10,111 people at high risk of a first fall. Given that the probability of falling in these groups ranged from 25 to 34%, the expected number of falls in these groups alone would be 2820 people.

**Table 2:** Logistic regression analysis of the 1^st^Fall risk groups against occurrence of a fall in Ontario, Manitoba, Alberta and British Colombia (validation samples)

	Ontario (*n* = 38,013)		Manitoba (*n* = 2739)		Alberta (*n* = 1226)		British Columbia (*n* = 9568)	
Risk group defined by 1^st^Fall	OR (95% CI)	N (%)	OR (95% CI)	N (%)	OR (95% CI)	N (%)	OR (95% CI)	N (%)
1 (reference)	–	1548 (4.07)	–	56 (2.05)	–	19 (1.55)	–	279 (2.92)
**2**	1.56 (1.29–1.87)	10,037 (26.40)	5.56 (1.34–23.12)	656 (23.96)	1.84 (0.41–8.24)	264 (21.53)	1.02 (0.73–1.44)	1448 (15.14)
**3**	2.20 (1.83–2.64)	12,633 (33.23)	6.23 (1.51–25.75)	1125 (41.09)	2.45 (0.56–10.75)	523 (42.66)	1.49 (1.08–2.06)	3665 (38.31)
**4**	3.10 (2.58–3.72)	9365 (24.64)	7.54 (1.82–31.28)	646 (23.59)	3.48 (0.79–15.36)	310 (25.29)	2.01 (1.45–2.78)	2847 (29.76)
**5**	4.02 (3.33–4.86)	4078 (10.73)	8.31 (1.96–35.23)	221 (8.07)	4.19 (0.92–19.15)	106 (8.65)	2.41 (1.72–3.38)	1179 (12.32)
**6**	6.28 (4.75–8.30)	352 (0.93)	9.72 (1.96–48.30)	34 (1.24)	8.50 (0.74–98.18)	4 (0.33)	3.27 (2.08–5.16)	148 (1.55)
c-statistic	0.60		0.55		0.58		0.58	

*OR*: Odds ratio, *CI*: Confidence Interv

### Fall-risk assessment algorithm validation

**Fig. 2:**
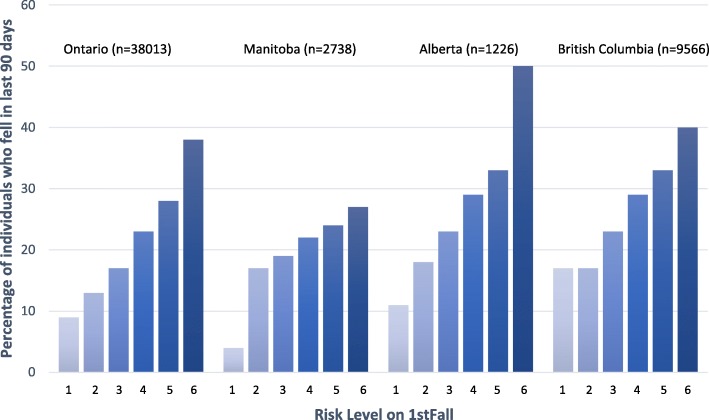
Percentage of individuals who fell in each risk group (total 6 groups) for Ontario, Manitoba, Alberta and British Colombia

The percentage of falls increased with each level defined by the algorithm (Fig. 2), and this trend was observed for all provinces. For each province, group 1 (lowest risk group) had the lowest percentage of falls and individuals in group 6 (highest risk group) had the highest percentage of falls. For Ontario, fall rates ranged from 9% (low risk group) to 38% (high risk group). For Manitoba, fall rates ranged from 4 to 27%, for Alberta they ranged from 11 to 50% and for British Columbia, they ranged from 17 to 40%.

Table 2 presents the results of the logistic regression analysis of the 1^st^Fall risk groups against the dependent variable fall status (0 falls versus 1 or more falls). For the Province of Ontario, odds ratio values increased as group level also increased from 1 (low risk) to 6 (high risk). For example, individuals in level 2 had an increased risk of falling as indicated by an odds ratio of 1.56 compared to in level 1. Further, individuals in level 4 had 3 times greater odds (odds ratio = 3.10) than those in level 1, and individuals in level 6 had 6 times greater odds (odds ratio = 6.28). Similar results were observed for Manitoba, Alberta and British Columbia, where the odds of sustaining a fall increased as risk level defined by the algorithm also increased.

## Discussion

This study developed an algorithm to predict first-time falls (1^st^Fall) in home care clients who had not fallen in the past 90 days. The 1^st^Fall algorithm identified 6 distinct risk categories for first time fallers from low to high risk. It also demonstrated predictive validity by performing equally well in four major provinces across Canada.

While previous studies have reported on models that predict falls in older adults, these studies typically used heterogeneous samples that included prior fallers [8–11]. A person’s fall history is the single best predictor of fall risk, [7] but it cannot be used in the identification of individuals at risk of falling for the first time. There is still a need for assessment tools that can predict the risk of a first fall onset. Most of the current literature has focused on individuals who are at high risk of falling (i.e. recurrent fallers) [7]. It would be preferable to identify elevated risk of falls prior to the event in order to reduce the rate of injuries that may themselves increase future fall risks.

Most published studies have generally assessed the relationship between different risk factors and falls with additive rather than interactive models [7, 24–26]. Decision tree analysis was preferred over conventional regression analysis for the following reasons. First, falls are multi-factorial phenomena, so the relationship between these factors is likely non-linear and may interact with each other to produce multiplicative patterns of risk. Regression analysis estimates the average effect of an independent variable on the outcome, while controlling for confounders. Decision trees provide a much more granular look at the data, with an ability to expose very small groups that share common characteristics, and that could be at high risk for falls [27]. Such small groups can easily be overlooked with regression analysis. Second, as the number of predictors increase in a regression model, the number of interaction effects that can be included also increases. The large number of possible interaction effects can be challenging to interpret and test for thoroughly. For instance, if there are 10 risk factors in a model, then there are (10*9)/2 = 45 potential interaction terms. Even though researchers typically only examine a smaller number of interactions, this restricts their ability to investigate the data fully [27, 28]. Third, regression models cannot assess individual coefficients alone because they are structured to evaluate them conditional to each other [28]. Last, decision trees can be excellent tools that provide a simple visual representation of complex associations, and identify sub-groups of at-risk individuals.

1^st^Fall identified combinations of 13 risk factors that predict first time falls in home care clients who have not fallen in the past 3 months. For example, an individual who had unsteady gait, cognitive impairment, Parkinsonism, unstable health patterns, and bladder incontinence had a high risk of falling for the first time. For policy makers, the 1^st^Fall algorithm may provide a standard assessment system to facilitate allocation of resources, improve efficiency of the health care system and reduce costs. For example, resources can be allocated to people who are at high risk of experiencing their first fall by providing physical and occupational therapy services, exercise classes, etc. Falls are very common causes of injury in older adults and are very costly to the health care system. The direct medical costs attributable to falls in older adults over the age of 65 is estimated at $32 billion in the United States [29] and $3.3 billion in Canada [30]. The 1^st^Fall algorithm can help reduce these costs by providing a proactive (rather than a reactive) approach to fall prevention. Furthermore, this prediction algorithm provides clinicians and case managers with a powerful tool to assess their clients and target preventative strategies. It can facilitate earlier identification of individuals who are at risk for falls and help develop personalized care plans. For example, unsteady gait may be targeted through referral to physical therapy and community-based exercise or balance programs. Urinary incontinence may be managed through education and pelvic floor muscle exercises. Cognitive impairment may be targeted through occupational therapy services to train specific abilities such as attention, memory or problem solving, or to teach compensatory strategies for daily living. Charts or schedules combined with education may improve management of medication [31]. As most of the factors in the algorithm are modifiable, if an intervention is implemented early enough, it may help prevent a fall onset. For instance, a recent network meta-analysis (including 54 randomized controlled trials, 41,596 participants) [32] demonstrated that exercise alone compared to usual care can reduce the odds of having an injurious fall by 50%. The efficacy of such interventions might further increase with appropriate targeting mechanisms like 1^st^Fall.

This paper highlights the advantages of having Minimum Data Sets such as those obtained through the interRAI suite of assessments, as they allow for the development of predictive algorithms that can inform decision making at the individual and population level. In countries that do not have the interRAI suite of assessments or similar datasets, the development of such algorithms may be a challenge. Alternative ways may be to perform secondary analysis of prospective cohort studies or existing medical records. However, a potential drawback of this approach is that the collected variables may not have undergone the same level of scrutiny or rigorous psychometric testing that is found with Minimum Data Sets.

A limitation of this study was that fall history was based on a recall period of 90 days. Further, the outcome falls was based mainly on self-reported data, which may have resulted in underreporting or recall bias. However, it is important to note that the evaluators who conduct the interviews are trained in obtaining the most accurate information possible.

## Conclusions

To our knowledge, this is the first study to predict a first time fall event in individuals who have not fallen in the past 90 days, using both decision tree analysis and a sample size of this magnitude. The RAI-HC, which was used to develop the algorithm, is a comprehensive standardized assessment that is being used in North America (Canada and multiple states in the U.S.), Europe, Asia and Oceania. 1^st^Fall has the potential to inform fall risk management and prevention of home care clients receiving services across the world. In Ontario, all adult home care clients who need home support or professional services in the community, are evaluated with the RAI-HC on admission and every 6 months thereafter. If implemented electronically, case managers who complete a RAI-HC assessment can automatically obtain a client’s risk classification, which in turn can initiate conversation around a client’s management plan. It can help case managers to engage in discussion with their clients about the factors that may be associated with a risk of falling and that may be modified through preventative actions. Therefore, future work will need to involve evaluation of the algorithm on an international scale and its application to different clinical contexts.

## Data Availability

Data used for secondary analysis in this project is not available publicly. De-identified datasets are maintained by interRAI with an understanding the data may be used for research purposes only by individuals and organizations who have a direct affiliation with the interRAI organization.

## Abbreviations

RAI-HC: Resident Assessment Instrument-Home Care
